# Supplementary material for: Pex30-dependent membrane contact sites maintain ER lipid homeostasis
Source: J Cell Biol. 2025 May 23;224(7):e202409039. doi: 10.1083/jcb.202409039 (PMC12101078; doi:10.1083/jcb.202409039)
Supplement: Table S1 — shows yeast strains used in this study. [file jcb_202409039_tables1.docx]

Table S1. Yeast strains used in this study.

| **Strain** | **Genotype** |
| --- | --- |
| BY4741 | *Mat a ura3∆0 HIS33∆1 leu2∆0 met15∆0* |
| yPC1658 | *Mat a ura3∆0 HIS33∆1 leu2∆0 met15∆0 hrd1∆::KAN* |
| yPC2008 | *Mat a ura3∆0 HIS33∆1 leu2∆0 met15∆0 asi1∆::KAN* |
| yPC3976 | *Mat a ura3∆0 HIS33∆1 leu2∆0 met15∆0 nem1∆::KAN* |
| yPC4062 | *Mat a ura3∆0 HIS33∆1 leu2∆0 met15∆0 vac8∆::KAN* |
| yPC5784 | *Mat a ura3∆0 HIS33∆1 leu2∆0 met15∆0 GPDpr-Cds1::KAN* |
| yPC6095 | *Mat a ura3∆0 HIS33∆1 leu2∆0 met15∆0 vps13∆::KAN* |
| yPC8018 | *Mat a ura3∆0 HIS33∆1 leu2∆0 met15∆0 hmg1∆::KAN* |
| yPC8806 | *Mat a ura3∆0 HIS33∆1 leu2∆0 met15∆0 pex28∆::KAN* |
| yPC8807 | *Mat a ura3∆0 HIS33∆1 leu2∆0 met15∆0 pex29∆::KAN* |
| yPC8808 | *Mat a ura3∆0 HIS33∆1 leu2∆0 met15∆0 pex30∆::KAN* |
| yPC8809 | *Mat a ura3∆0 HIS33∆1 leu2∆0 met15∆0 pex31∆::KAN* |
| yPC8810 | *Mat a ura3∆0 HIS33∆1 leu2∆0 met15∆0 pex32∆::KAN* |
| yPC10657 | *Mat a ura3∆0 HIS33∆1 leu2∆0 met15∆0* *Pex30-mNG::HIS3* |
| yPC10865 | *Mat a ura3∆0 HIS33∆1 leu2∆0 met15∆0* *Pex30-mNG::KAN Nvj1-tdTomato::HIS3* |
| yPC10881 | *Mat a ura3∆0 HIS33∆1 leu2∆0 met15∆0 Tsc13-sfGFP-URA3 Vph1-tdTomato-HIS3* |
| yPC11802 | *Mat a ura3∆0 HIS33∆1 leu2∆0 met15∆0 mCherry-PTS1::LEU2(HO locus)* |
| yPC11803 | *Mat a ura3∆0 HIS33∆1 leu2∆0 met15∆0 pex30Δ::KAN mCherry-PTS1::LEU2(HO locus)* |
| yPC11840 | *Mat ? ura3∆0 HIS33∆1 leu2∆0 met15∆0 Pex29-mNG::HYGB Nvj1-tdTomato::HIS3* |
| yPC11843 | *Mat ? ura3∆0 HIS33∆1 leu2∆0 met15∆0 Pex30-mNG::HYGB Nvj1-tdTomato::HIS3* |
| yPC11876 | *Mat a ura3∆0 HIS33∆1 leu2∆0 met15∆0 Pex28-13xMyc::HIS3 Pex29-V5::(CRISPR) Pex32-3xHA::KAN* |
| yPC11933 | *Mat a ura3∆0 HIS33∆1 leu2∆0 met15∆0 inp1∆::KAN* |
| yPC11976 | *Mat a ura3∆0 HIS33∆1 leu2∆0 met15∆0 Pex28-13xMyc::HIS3 Pex29-V5::(CRISPR) Pex32-3xHA::KAN Pex30(284-408∆)::(CRISPR)* |
| yPC12061 | *Mat a ura3∆0 HIS33∆1 leu2∆0 met15∆0 nvj1∆::KAN* |
| yPC12063 | *Mat a ura3∆0 HIS33∆1 leu2∆0 met15∆0 Pex30-mNG::KAN Nvj1-tdTomato::HIS3 pex29∆::(CRISPR)* |
| yPC12181 | *Mat a ura3∆0 HIS33∆1 leu2∆0 met15∆0 Pex28-13xMyc::HIS3 Pex29-V5::(CRISPR) Pex32-3xHA::KAN pex30∆::ZeoR* |
| yPC12234 | *Mat a ura3∆0 HIS33∆1 leu2∆0 met15∆0 Pex32(1-308)-3HA::KAN Pex28-13myc::HIS3* |
| yPC12254 | *Mat a ura3∆0 HIS33∆1 leu2∆0 met15∆0 Pex32-mNG::KAN Pex30(Δ284-408) PTS1-mCherry* |
| yPC12255 | *Mat a ura3∆0 HIS33∆1 leu2∆0 met15∆0 Pex32-mNG::KAN Pex30(Δ415-513) PTS1-mCherry* |
| yPC12279 | *Mat a ura3∆0 HIS33∆1 leu2∆0 met15∆0 Pex32-3HA::KAN Pex29-V5::(CRISPR) Pex28(467-573Δ)-13myc::HIS3(CRISPR)* |
| yPC12282 | *Mat a ura3∆0 HIS33∆1 leu2∆0 met15∆0 Pex32-3HA::KAN Pex29(351-470Δ)-V5(CRISPR) Pex28-13myc::HIS3* |
| yPC12287 | *Mat a ura3∆0 HIS33∆1 leu2∆0 met15∆0 Pex30(284-408Δ)-mNG::HIS3(CRISPR) Nvj1-tdTomato::KAN* |
| yPC12304 | *Mat a ura3∆0 HIS33∆1 leu2∆0 met15∆0 Pex29(351-408Δ)-mNG(CRISPR) Nvj1-tdTomato::HIS3* |
| yPC1231 | *Mat ? ura3∆0 HIS33∆1 leu2∆0 met15∆0 pex28∆::KAN pex29∆::HYGB* |
| yPC12342 | *Mat a ura3∆0 HIS33∆1 leu2∆0 met15∆0 Pex32-3HA::HYGB Pex29-V5::(CRISPR) Pex28-13myc::HIS3 Pex30(446A)::(CRISPR)* |
| yPC12345 | *Mat a ura3∆0 HIS33∆1 leu2∆0 met15∆0 Pex30(446A)-mNG::HYGB(CRISPR) Nvj1-tdTomato::HIS3* |
| yPC12347 | *Mat a ura3∆0 HIS33∆1 leu2∆0 met15∆0 Pex30(1-410)-mNG::ZeoR Nvj1-tdTomato::His* |
| yPC12554 | *Mat a ura3∆0 HIS33∆1 leu2∆0 met15∆0 Pex30(S446D)-mNG::HYGB(CRISPR) Nvj1-tdtomato::HIS3* |
| yPC12578 | *Mat a ura3∆0 HIS33∆1 leu2∆0 met15∆0 Pex32-3HA::KAN Pex29-V5::(CRISPR) Pex28-13myc::HIS3 Pex30(S446A)::(CRISPR) mCherry-PTS1::LEU2(HO locus)* |
| yPC12579 | *Mat a ura3∆0 HIS33∆1 leu2∆0 met15∆0 Pex32-3HA::KAN Pex29-V5::(CRISPR) Pex28-13myc::HIS3 Pex30(446D) mCherry-PTS1::LEU2(HO locus)* |
| yPC12581 | *Mat a ura3∆0 HIS33∆1 leu2∆0 met15∆0 Pex32-3HA::HYGB Pex29-V5::(CRISPR) Pex28-13myc::HIS3 Pex30(S446D)::(CRISPR)* |
| yPC12591 | *Mat a ura3∆0 HIS33∆1 leu2∆0 met15∆0 Pex32-mNG::KAN Inp1-mCherry::URA3* |
| yPC12592 | *Mat a ura3∆0 HIS33∆1 leu2∆0 met15∆0 Pex32-mNG::KAN Inp1-mCherry::URA3 pex30∆::(CRISPR)* |
| yPC12593 | *Mat a ura3∆0 HIS33∆1 leu2∆0 met15∆0 Pex32-mNG::KAN Inp1-mCherry::URA3 Pex30(284-408∆)::(CRISPR)* |
| yPC12594 | *Mat a ura3∆0 HIS33∆1 leu2∆0 met15∆0 Pex32-mNG::KAN Inp1-mCherry::URA3 Pex30(415-513∆)::(CRISPR)* |
| yPC12595 | *Mat a ura3∆0 HIS33∆1 leu2∆0 met15∆0 Pex32(307-413Δ)-mNG Inp1-mCherry::URA3* |
| yPC12712 | *Mat a ura3∆0 HIS33∆1 leu2∆0 met15∆0 Mam3-myc-3C-3xFLAG::KAN* |
| yPC12713 | *Mat a ura3∆0 HIS33∆1 leu2∆0 met15∆0 Mam3-myc-3C-3xFLAG::KAN pex30∆::HIS3* |
| yPC12714 | *Mat a ura3∆0 HIS33∆1 leu2∆0 met15∆0 Mam3-myc-3C-3xFLAG::KAN pex29∆::HYGB* |
| yPC12746 | *Mat a ura3∆0 HIS33∆1 leu2∆0 met15∆0 Pex3-mNG::KAN Inp1-mCherry::HIS* |
| yPC12747 | *Mat a ura3∆0 HIS33∆1 leu2∆0 met15∆0 Pex3-mNG::KAN Inp1-mCherry::HIS pex30::KAN* |
| yPC12780 | *Mat a ura3∆0 HIS33∆1 leu2∆0 met15∆0 Pex32-mNG:KAN: Inp1-mCherry::URA3 pex29Δ::(CRISPR)* |
| yPC12785 | *Mat a ura3∆0 HIS33∆1 leu2∆0 met15∆0 yPC12554 Pex30(S446D)-mNG::HYGB Nvj1-tdtomato::HIS3 pex29Δ::KAN* |
| yPC12786 | *Mat a ura3∆0 HIS33∆1 leu2∆0 met15∆0 yPC12554 Pex30(S446D,284-408Δ)-mNG::HYGB Nvj1-tdtomato::HIS3* |
| yPC12791 | *Mat a ura3∆0 HIS33∆1 leu2∆0 met15∆0 pex31∆::KAN pex30∆::(CRISPR)* |
| yPC12972 | *Mat a ura3∆0 HIS33∆1 leu2∆0 met15∆0 Pex30(284-408∆)::(CRISPR)* |
| yPC12973 | *Mat a ura3∆0 HIS33∆1 leu2∆0 met15∆0 Pex30(Δ415-513)::(CRISPR)* |
| yPC12974 | *Mat a ura3∆0 HIS33∆1 leu2∆0 met15∆0 GPDpr-Cds1::KAN pex30∆::HIS* |
| yPC12975 | *Mat a ura3∆0 HIS33∆1 leu2∆0 met15∆0 pex3∆::KAN* |
| yPC12986 | *Mat a ura3∆0 HIS33∆1 leu2∆0 met15∆0 Pex30(I301A, K304A, F392A, Y395A)::(CRISPR)* |
| yPC12992 | *Mat a ura3∆0 HIS33∆1 leu2∆0 met15∆0 Pex28-13xMyc::HIS3 Pex29-V5::(CRISPR) Pex32-3xHA::KAN Pex30(W298A, I301A, K304A, F392A, Y395A)::(CRISPR)* |
| yPC12999 | *Mat ? ura3∆0 HIS33∆1 leu2∆0 met15∆0 Pex30(I301A, K304A, F392A, Y395A)-mNG::HYGB Nvj1-tdTomato::HIS3* |
| yPC13043 | *Mat a ura3∆0 HIS33∆1 leu2∆0 met15∆0 Pex32-mNG::KAN Inp1-mCherry::URA3 Pex30(I301A, K304A, F392A, Y395A)* |
| yPC13110 | *Mat a ura3∆0 HIS33∆1 leu2∆0 met15∆0 Tsc13-sfGFP-URA3 Vph1-tdTomato-HIS3 Pex30(S446D)::CRISPR* |
| yPC13111 | *Mat a ura3∆0 HIS33∆1 leu2∆0 met15∆0 Tsc13-sfGFP-URA3 Vph1-tdTomato-HIS3 pex30::KAN* |
