# Supplementary material for: Pex30-dependent membrane contact sites maintain ER lipid homeostasis
Source: J Cell Biol. 2025 May 23;224(7):e202409039. doi: 10.1083/jcb.202409039 (PMC12101078; doi:10.1083/jcb.202409039)
Supplement: Table S2 — shows plasmids used in this study. [file jcb_202409039_tables2.docx]

Table S2. Plasmids used in this study.

| **Plasmid** | **Recombinant DNA** |
| --- | --- |
| pPC220 | *pFA6a-3xHA-KANMX6* |
| pPC225 | *pFA6a-13xMyc-HIS3MX6* |
| pPC653 | *pFA6a-mCherry-HIS3MX6* |
| pPC655 | *pFA6a-tdTomato-HIS3MX6* |
| pPC852 | *pRS416-ADHpr-mRFP-FYVE(EEA1)* |
| pPC853 | *pRS415-GFPpr-mCherry-2xPH(Osh2)* |
| pPC1034 | *pRS416-GFP-Lact-C2* |
| pPC1463 | *ADH1p-PTS1-mCherry-LEU* |
| pPC1516 | *pFA6a-mNG-KANMX6* |
| pPC1517 | *pFA6a-mNG-HIS3MX6* |
| pPC1702 | *YEplac181-humanPKD(136-343)-GFP-Ubc6TM* |
| pPC1738 | *pML107-Pex30-gRNA1 (GATGGGTCGACAAGACATGG)* |
| pPC1753 | *pML107-Pex28-gRNA3 (AGAGCACAGCAATTCCCACC)* |
| pPC1755 | *pML107-Pex29-gRNA2 (CTAGTTTGAGAATATACCAG)* |
| pPC1873 | *pWS176 - ZeoR* |
| pPC2073 | *pML107-Pex30-gRNA17 (TAACCGCTGAGCAAGAGCTT)* |
| pPC2074 | *pK27-Pex30(284-412)-HA* |
| pPC2075 | *pK27-Pex29(350-472)-V5* |
| pPC2076 | *pK27-Pex31(273-408)-Flag* |
| pPC2077 | *pK27-Pex28(465-573)-V5* |
| pPC2078 | *pK27-Pex32(305-413)-Flag* |
| pPC2080 | *pK27-Opi1(103-189)-Flag* |
| pPC2616 | *pDA179-mCherry-D4H* |
| pPC2622 | *pK27-Dysferlin(939-1066)-V5* |
| pPC2628 | *YEplac181-Spo20(51-91)-GFP-Ubc6TM* |
| pPC2634 | *pFA6a-myc-3C-3xFLAG-kanMX6* |
| pPC2636 | *pML107-Pex30-gRNA18 (TCTTCTTGTATATTTAGAAA)* |
| pPC2637 | *pML107-Pex30-gRNA19 (TCTTCTTGTATATTTAGAAA)* |
