# Supplementary material for: Pex30-dependent membrane contact sites maintain ER lipid homeostasis
Source: J Cell Biol. 2025 May 23;224(7):e202409039. doi: 10.1083/jcb.202409039 (PMC12101078; doi:10.1083/jcb.202409039)
Supplement: Table S3 — shows posttranslational modifications reported for Pex30. [file jcb_202409039_tables3.docx]

Table S3. Post-translation modifications reported for Pex30.

| **Residue** | **Modification** | **Reference** |
| --- | --- | --- |
| S2 | Phosphorylation | [(Hu et al., 2019; Lanz et al., 2021)](https://sciwheel.com/work/citation?ids=16741373,10427390&pre=&pre=&suf=&suf=&sa=0,0&dbf=0&dbf=0) |
| N4 | N-glycosylation | [(Zielinska et al., 2012)](https://sciwheel.com/work/citation?ids=1587359&pre=&suf=&sa=0&dbf=0) |
| K37 | Ubiquitination | [(Swaney et al., 2013)](https://sciwheel.com/work/citation?ids=1240375&pre=&suf=&sa=0&dbf=0) |
| S42 | Phosphorylation | [(Helbig et al., 2010; Lanz et al., 2021)](https://sciwheel.com/work/citation?ids=2118022,10427390&pre=&pre=&suf=&suf=&sa=0,0&dbf=0&dbf=0) |
| S45 | Phosphorylation | [(Hu et al., 2019; Lanz et al., 2021; Zhou et al., 2021)](https://sciwheel.com/work/citation?ids=16741373,10427390,10338466&pre=&pre=&pre=&suf=&suf=&suf=&sa=0,0,0&dbf=0&dbf=0&dbf=0) |
| S52 | Phosphorylation | [(Albuquerque et al., 2008; Hu et al., 2019; Lanz et al., 2021; Pultz et al., 2012; Swaney et al., 2013; Zhou et al., 2021)](https://sciwheel.com/work/citation?ids=145043,16741373,10427390,16741402,1240375,10338466&pre=&pre=&pre=&pre=&pre=&pre=&suf=&suf=&suf=&suf=&suf=&suf=&sa=0,0,0,0,0,0&dbf=0&dbf=0&dbf=0&dbf=0&dbf=0&dbf=0) |
| T60 | Phosphorylation | [(Lanz et al., 2021)](https://sciwheel.com/work/citation?ids=10427390&pre=&suf=&sa=0&dbf=0) |
| S61 | Phosphorylation | [(Albuquerque et al., 2008; Hu et al., 2019)](https://sciwheel.com/work/citation?ids=145043,16741373&pre=&pre=&suf=&suf=&sa=0,0&dbf=0&dbf=0) |
| S280 | Phosphorylation | [(Lanz et al., 2021)](https://sciwheel.com/work/citation?ids=10427390&pre=&suf=&sa=0&dbf=0) |
| T288 | Phosphorylation | [(Holt et al., 2009)](https://sciwheel.com/work/citation?ids=145170&pre=&suf=&sa=0&dbf=0) |
| Y289 | Phosphorylation | [(Holt et al., 2009)](https://sciwheel.com/work/citation?ids=145170&pre=&suf=&sa=0&dbf=0) |
| T410 | Phosphorylation | [(Helbig et al., 2010)](https://sciwheel.com/work/citation?ids=2118022&pre=&suf=&sa=0&dbf=0) |
| S411 | Phosphorylation | [(Helbig et al., 2010)](https://sciwheel.com/work/citation?ids=2118022&pre=&suf=&sa=0&dbf=0) |
| S416 | Phosphorylation | [(Gnad et al., 2009; Holt et al., 2009; Hu et al., 2019; Lanz et al., 2021; Soufi et al., 2009; Zhou et al., 2021)](https://sciwheel.com/work/citation?ids=747333,145170,16741373,10427390,1155662,10338466&pre=&pre=&pre=&pre=&pre=&pre=&suf=&suf=&suf=&suf=&suf=&suf=&sa=0,0,0,0,0,0&dbf=0&dbf=0&dbf=0&dbf=0&dbf=0&dbf=0) |
| S420 | Phosphorylation | [(Albuquerque et al., 2008; Holt et al., 2009; Hu et al., 2019; Lanz et al., 2021; MacGilvray et al., 2020; Swaney et al., 2013; Zhou et al., 2021)](https://sciwheel.com/work/citation?ids=145043,145170,16741373,10427390,12383621,1240375,10338466&pre=&pre=&pre=&pre=&pre=&pre=&pre=&suf=&suf=&suf=&suf=&suf=&suf=&suf=&sa=0,0,0,0,0,0,0&dbf=0&dbf=0&dbf=0&dbf=0&dbf=0&dbf=0&dbf=0) |
| S424 | Phosphorylation | [(Albuquerque et al., 2008; Gnad et al., 2009; Holt et al., 2009; Hu et al., 2019; Lanz et al., 2021; MacGilvray et al., 2020; Soufi et al., 2009; Soulard et al., 2010; Swaney et al., 2013; Zhou et al., 2021)](https://sciwheel.com/work/citation?ids=145043,747333,145170,16741373,10427390,12383621,1155662,2588829,1240375,10338466&pre=&pre=&pre=&pre=&pre=&pre=&pre=&pre=&pre=&pre=&suf=&suf=&suf=&suf=&suf=&suf=&suf=&suf=&suf=&suf=&sa=0,0,0,0,0,0,0,0,0,0&dbf=0&dbf=0&dbf=0&dbf=0&dbf=0&dbf=0&dbf=0&dbf=0&dbf=0&dbf=0) |
| S434 | Phosphorylation | [(Hu et al., 2019; Lanz et al., 2021; Swaney et al., 2013)](https://sciwheel.com/work/citation?ids=16741373,10427390,1240375&pre=&pre=&pre=&suf=&suf=&suf=&sa=0,0,0&dbf=0&dbf=0&dbf=0) |
| Y435 | Phosphorylation | [(Lanz et al., 2021)](https://sciwheel.com/work/citation?ids=10427390&pre=&suf=&sa=0&dbf=0) |
| S446 | Phosphorylation | [(Hu et al., 2019; Lanz et al., 2021)](https://sciwheel.com/work/citation?ids=16741373,10427390&pre=&pre=&suf=&suf=&sa=0,0&dbf=0&dbf=0) |
| S483 | Phosphorylation | [(Zhou et al., 2021)](https://sciwheel.com/work/citation?ids=10338466&pre=&suf=&sa=0&dbf=0) |
| S484 | Phosphorylation | [(Albuquerque et al., 2008; Holt et al., 2009; Hu et al., 2019; Lanz et al., 2021; Zhou et al., 2021)](https://sciwheel.com/work/citation?ids=145043,145170,16741373,10427390,10338466&pre=&pre=&pre=&pre=&pre=&suf=&suf=&suf=&suf=&suf=&sa=0,0,0,0,0&dbf=0&dbf=0&dbf=0&dbf=0&dbf=0) |
| T485 | Phosphorylation | [(Albuquerque et al., 2008; Hu et al., 2019; Lanz et al., 2021; Zhou et al., 2021)](https://sciwheel.com/work/citation?ids=145043,16741373,10427390,10338466&pre=&pre=&pre=&pre=&suf=&suf=&suf=&suf=&sa=0,0,0,0&dbf=0&dbf=0&dbf=0&dbf=0) |
| K508 | Ubiquitination | [(Swaney et al., 2013)](https://sciwheel.com/work/citation?ids=1240375&pre=&suf=&sa=0&dbf=0) |
| S519 | Phosphorylation | [(Hu et al., 2019)](https://sciwheel.com/work/citation?ids=16741373&pre=&suf=&sa=0&dbf=0) |
| Non-assigned | Ubiquitination | [(Kolawa et al., 2013; Peng et al., 2003; Phillips et al., 2013)](https://sciwheel.com/work/citation?ids=4435628,80979,1206915&pre=&pre=&pre=&suf=&suf=&suf=&sa=0,0,0&dbf=0&dbf=0&dbf=0) |

**References**

[Albuquerque, C. P., Smolka, M. B., Payne, S. H., Bafna, V., Eng, J., & Zhou, H. (2008). A multidimensional chromatography technology for in-depth phosphoproteome analysis. *Molecular & Cellular Proteomics*, *7*(7), 1389–1396. https://doi.org/10.1074/mcp.M700468-MCP200](https://sciwheel.com/work/bibliography/145043)

[Gnad, F., de Godoy, L. M. F., Cox, J., Neuhauser, N., Ren, S., Olsen, J. V., & Mann, M. (2009). High-accuracy identification and bioinformatic analysis of in vivo protein phosphorylation sites in yeast. *Proteomics*, *9*(20), 4642–4652. https://doi.org/10.1002/pmic.200900144](https://sciwheel.com/work/bibliography/747333)

[Helbig, A. O., Rosati, S., Pijnappel, P. W. W. M., van Breukelen, B., Timmers, M. H. T. H., Mohammed, S., Slijper, M., & Heck, A. J. R. (2010). Perturbation of the yeast N-acetyltransferase NatB induces elevation of protein phosphorylation levels. *BMC Genomics*, *11*, 685. https://doi.org/10.1186/1471-2164-11-685](https://sciwheel.com/work/bibliography/2118022)

[Holt, L. J., Tuch, B. B., Villén, J., Johnson, A. D., Gygi, S. P., & Morgan, D. O. (2009). Global analysis of Cdk1 substrate phosphorylation sites provides insights into evolution. *Science*, *325*(5948), 1682–1686. https://doi.org/10.1126/science.1172867](https://sciwheel.com/work/bibliography/145170)

[Hu, Z., Raucci, S., Jaquenoud, M., Hatakeyama, R., Stumpe, M., Rohr, R., Reggiori, F., De Virgilio, C., & Dengjel, J. (2019). Multilayered control of protein turnover by TORC1 and atg1. *Cell Reports*, *28*(13), 3486-3496.e6. https://doi.org/10.1016/j.celrep.2019.08.069](https://sciwheel.com/work/bibliography/16741373)

[Kolawa, N., Sweredoski, M. J., Graham, R. L. J., Oania, R., Hess, S., & Deshaies, R. J. (2013). Perturbations to the ubiquitin conjugate proteome in yeast δubx mutants identify Ubx2 as a regulator of membrane lipid composition. *Molecular & Cellular Proteomics*, *12*(10), 2791–2803. https://doi.org/10.1074/mcp.M113.030163](https://sciwheel.com/work/bibliography/4435628)

[Lanz, M. C., Yugandhar, K., Gupta, S., Sanford, E. J., Faça, V. M., Vega, S., Joiner, A. M. N., Fromme, J. C., Yu, H., & Smolka, M. B. (2021). In-depth and 3-dimensional exploration of the budding yeast phosphoproteome. *EMBO Reports*, *22*(2), e51121. https://doi.org/10.15252/embr.202051121](https://sciwheel.com/work/bibliography/10427390)

[MacGilvray, M. E., Shishkova, E., Place, M., Wagner, E. R., Coon, J. J., & Gasch, A. P. (2020). Phosphoproteome Response to Dithiothreitol Reveals Unique Versus Shared Features of Saccharomyces cerevisiae Stress Responses. *Journal of Proteome Research*, *19*(8), 3405–3417. https://doi.org/10.1021/acs.jproteome.0c00253](https://sciwheel.com/work/bibliography/12383621)

[Peng, J., Schwartz, D., Elias, J. E., Thoreen, C. C., Cheng, D., Marsischky, G., Roelofs, J., Finley, D., & Gygi, S. P. (2003). A proteomics approach to understanding protein ubiquitination. *Nature Biotechnology*, *21*(8), 921–926. https://doi.org/10.1038/nbt849](https://sciwheel.com/work/bibliography/80979)

[Phillips, A. H., Zhang, Y., Cunningham, C. N., Zhou, L., Forrest, W. F., Liu, P. S., Steffek, M., Lee, J., Tam, C., Helgason, E., Murray, J. M., Kirkpatrick, D. S., Fairbrother, W. J., & Corn, J. E. (2013). Conformational dynamics control ubiquitin-deubiquitinase interactions and influence in vivo signaling. *Proceedings of the National Academy of Sciences of the United States of America*, *110*(28), 11379–11384. https://doi.org/10.1073/pnas.1302407110](https://sciwheel.com/work/bibliography/1206915)

[Pultz, D., Bennetzen, M. V., Rødkær, S. V., Zimmermann, C., Enserink, J. M., Andersen, J. S., & Færgeman, N. J. (2012). Global mapping of protein phosphorylation events identifies Ste20, Sch9 and the cell-cycle regulatory kinases Cdc28/Pho85 as mediators of fatty acid starvation responses in Saccharomyces cerevisiae. *Molecular Biosystems*, *8*(3), 796–803. https://doi.org/10.1039/c2mb05356j](https://sciwheel.com/work/bibliography/16741402)

[Soufi, B., Kelstrup, C. D., Stoehr, G., Fröhlich, F., Walther, T. C., & Olsen, J. V. (2009). Global analysis of the yeast osmotic stress response by quantitative proteomics. *Molecular Biosystems*, *5*(11), 1337–1346. https://doi.org/10.1039/b902256b](https://sciwheel.com/work/bibliography/1155662)

[Soulard, A., Cremonesi, A., Moes, S., Schütz, F., Jenö, P., & Hall, M. N. (2010). The rapamycin-sensitive phosphoproteome reveals that TOR controls protein kinase A toward some but not all substrates. *Molecular Biology of the Cell*, *21*(19), 3475–3486. https://doi.org/10.1091/mbc.E10-03-0182](https://sciwheel.com/work/bibliography/2588829)

[Swaney, D. L., Beltrao, P., Starita, L., Guo, A., Rush, J., Fields, S., Krogan, N. J., & Villén, J. (2013). Global analysis of phosphorylation and ubiquitylation cross-talk in protein degradation. *Nature Methods*, *10*(7), 676–682. https://doi.org/10.1038/nmeth.2519](https://sciwheel.com/work/bibliography/1240375)

[Zhou, X., Li, W., Liu, Y., & Amon, A. (2021). Cross-compartment signal propagation in the mitotic exit network. *ELife*, *10*. https://doi.org/10.7554/eLife.63645](https://sciwheel.com/work/bibliography/10338466)

[Zielinska, D. F., Gnad, F., Schropp, K., Wiśniewski, J. R., & Mann, M. (2012). Mapping N-glycosylation sites across seven evolutionarily distant species reveals a divergent substrate proteome despite a common core machinery. *Molecular Cell*, *46*(4), 542–548. https://doi.org/10.1016/j.molcel.2012.04.031](https://sciwheel.com/work/bibliography/1587359)
