# Supplementary figures and images for: Pex30-dependent membrane contact sites maintain ER lipid homeostasis
Source: J Cell Biol. 2025 May 23;224(7):e202409039. doi: 10.1083/jcb.202409039 (PMC12101078; doi:10.1083/jcb.202409039)

F

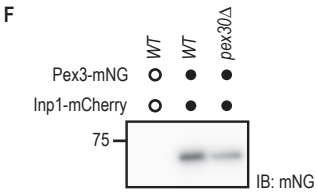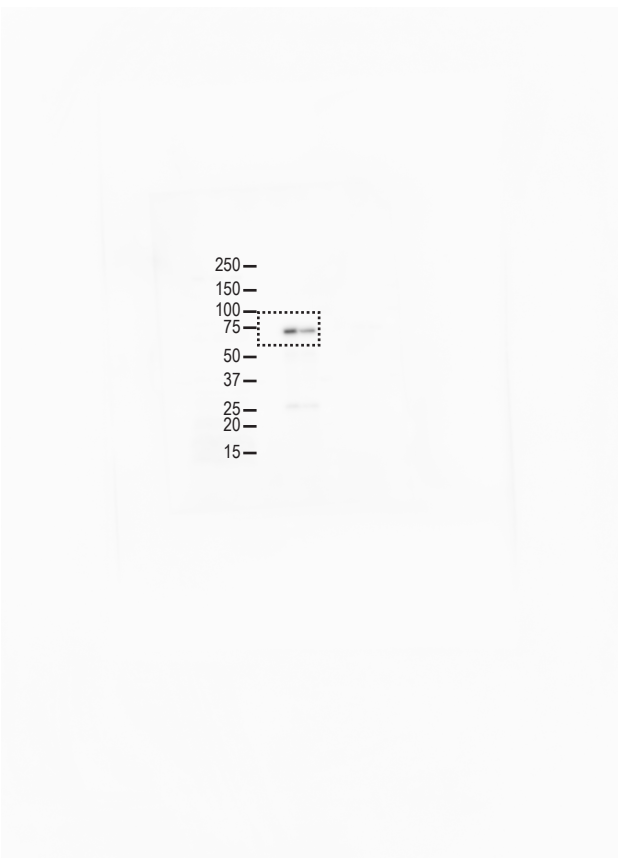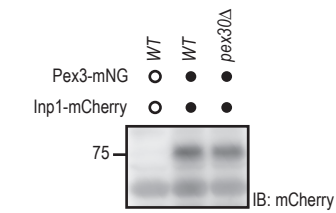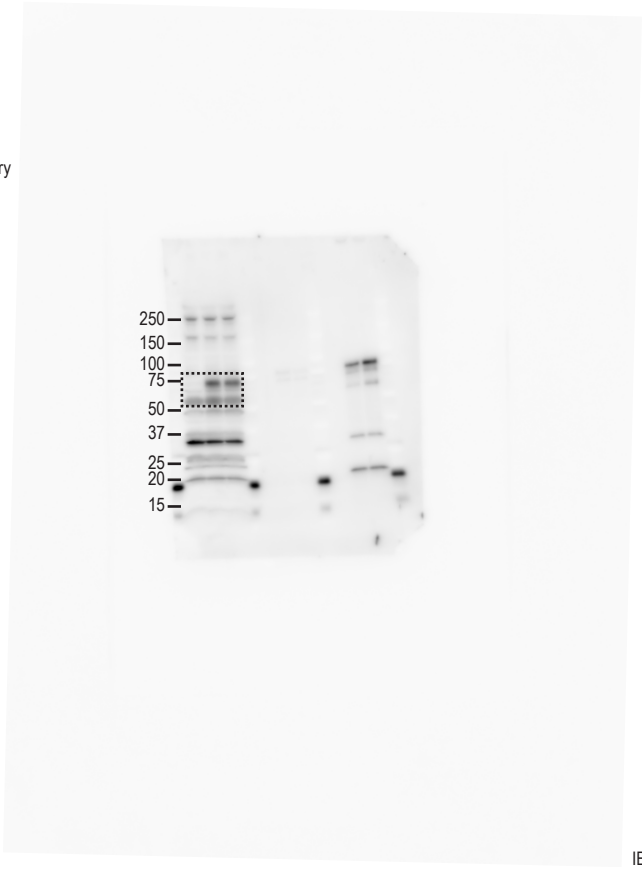

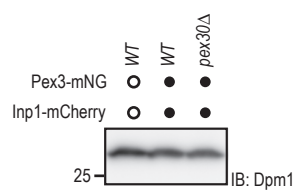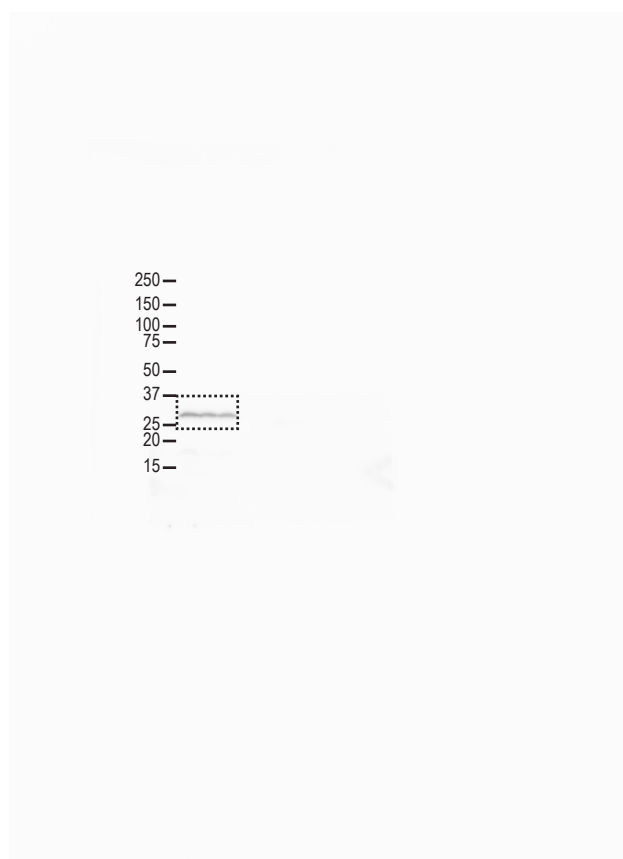

Supplement: SourceData F1 — is the source file for Fig. 1. [file jcb_202409039_sourcedataf1.pdf]

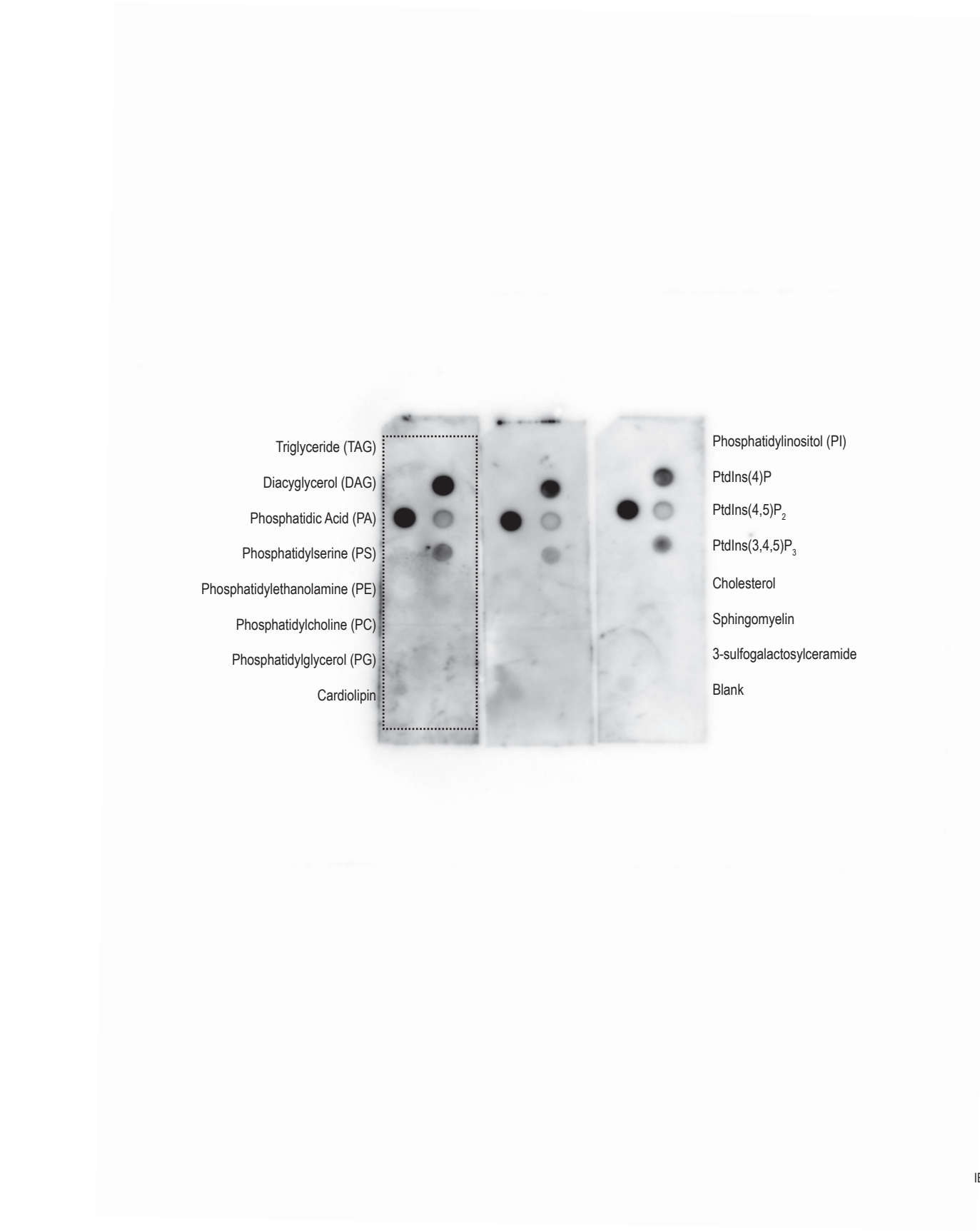

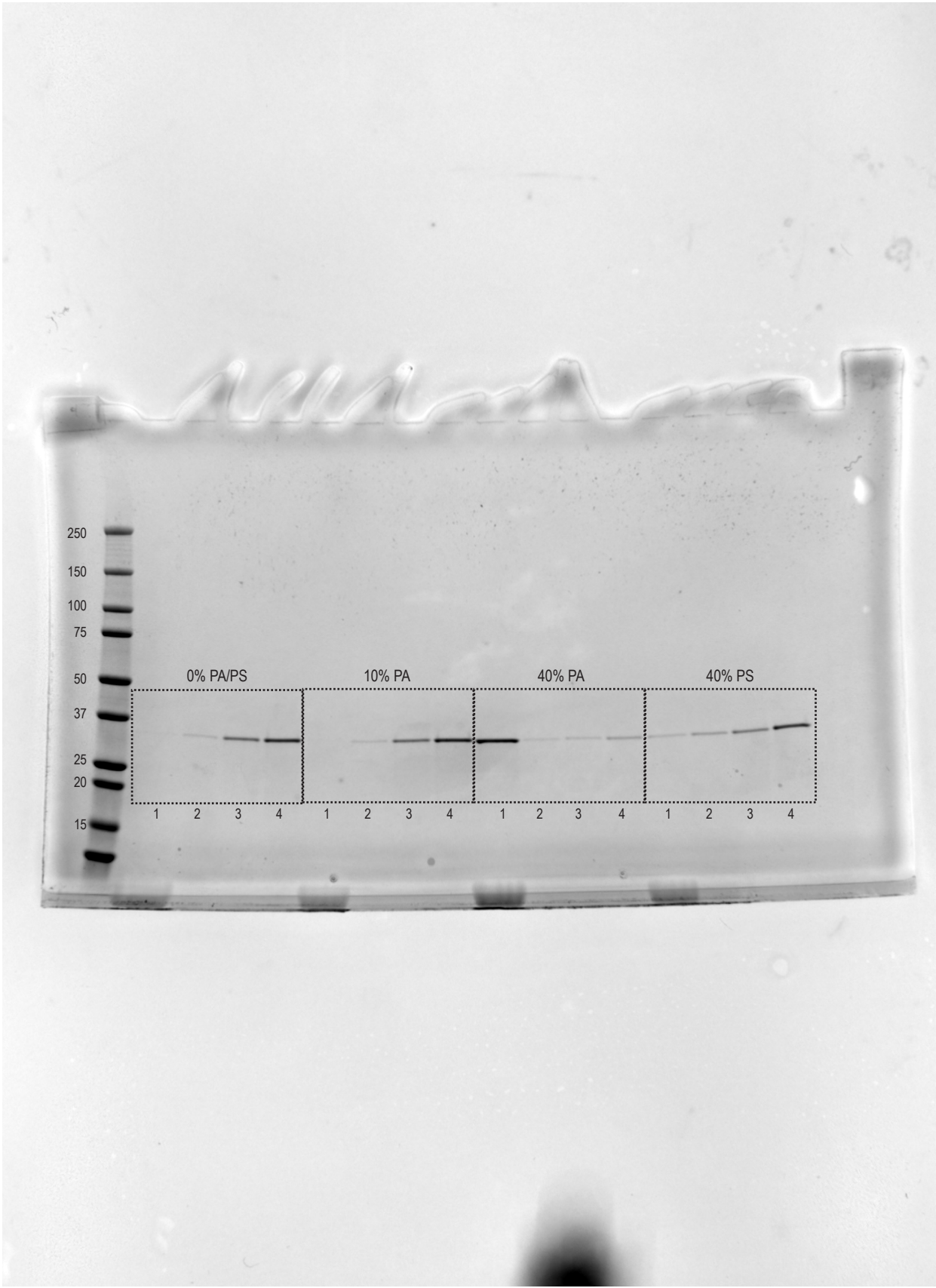

Coomassie

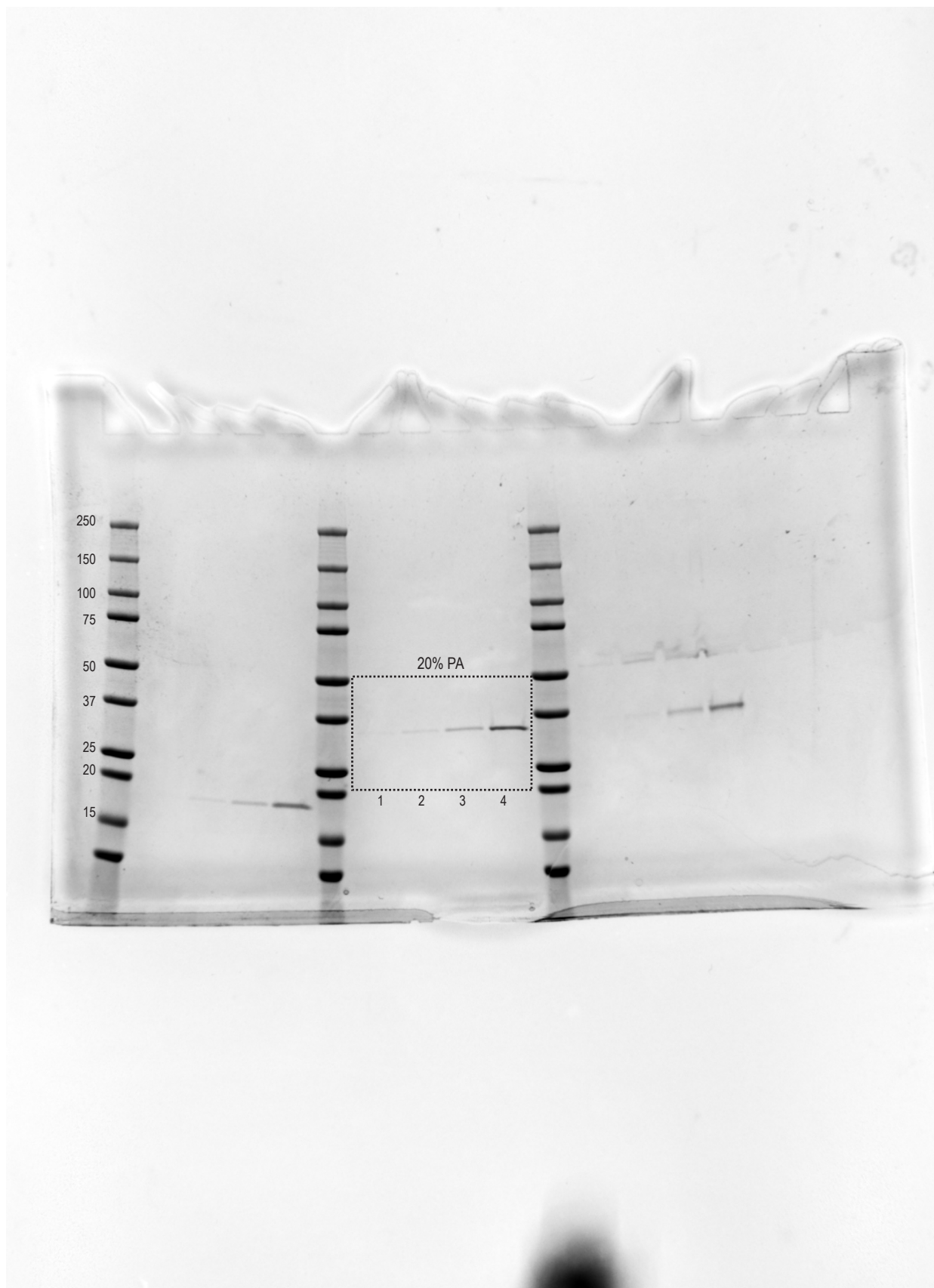

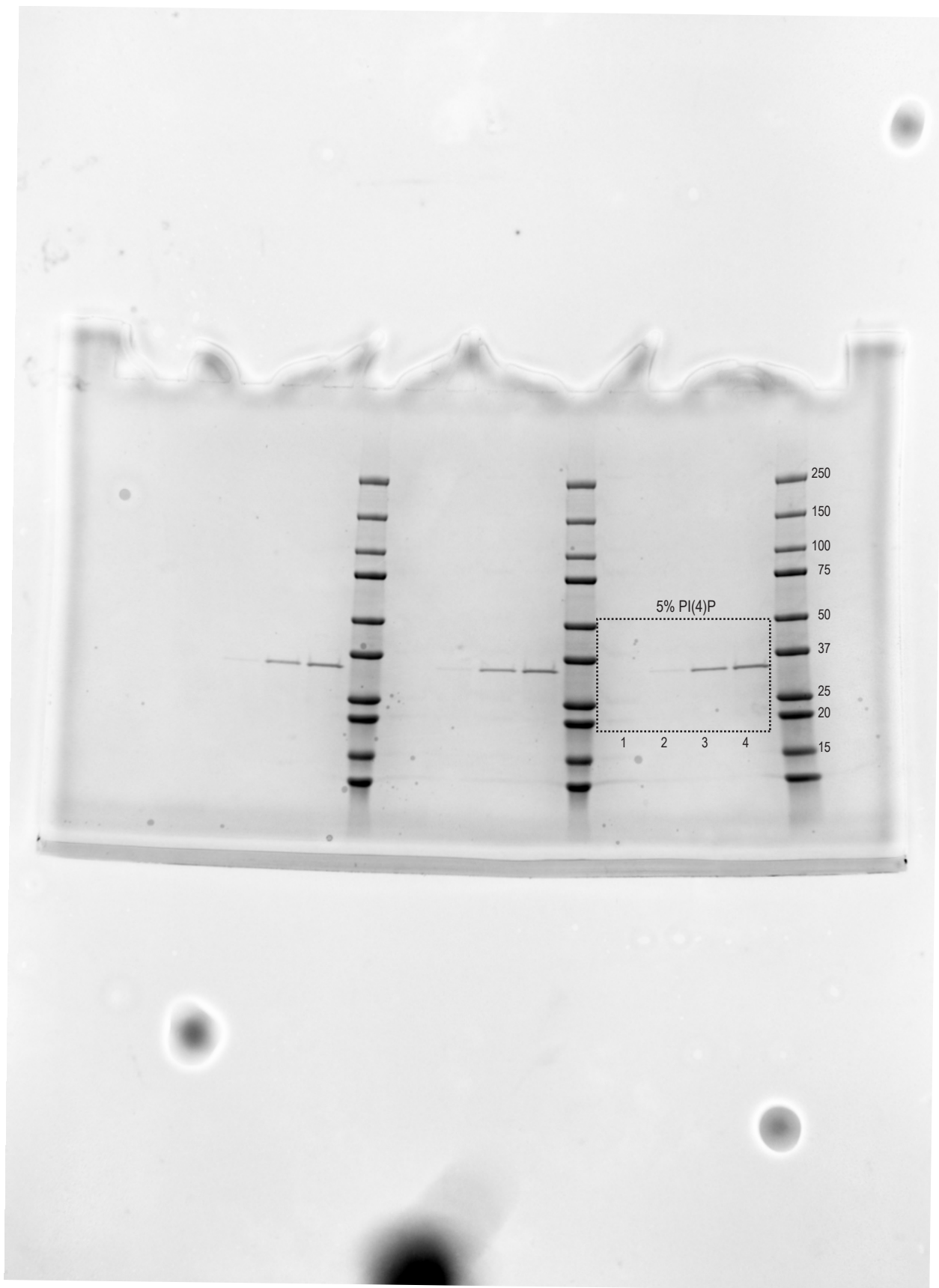

Coomassie

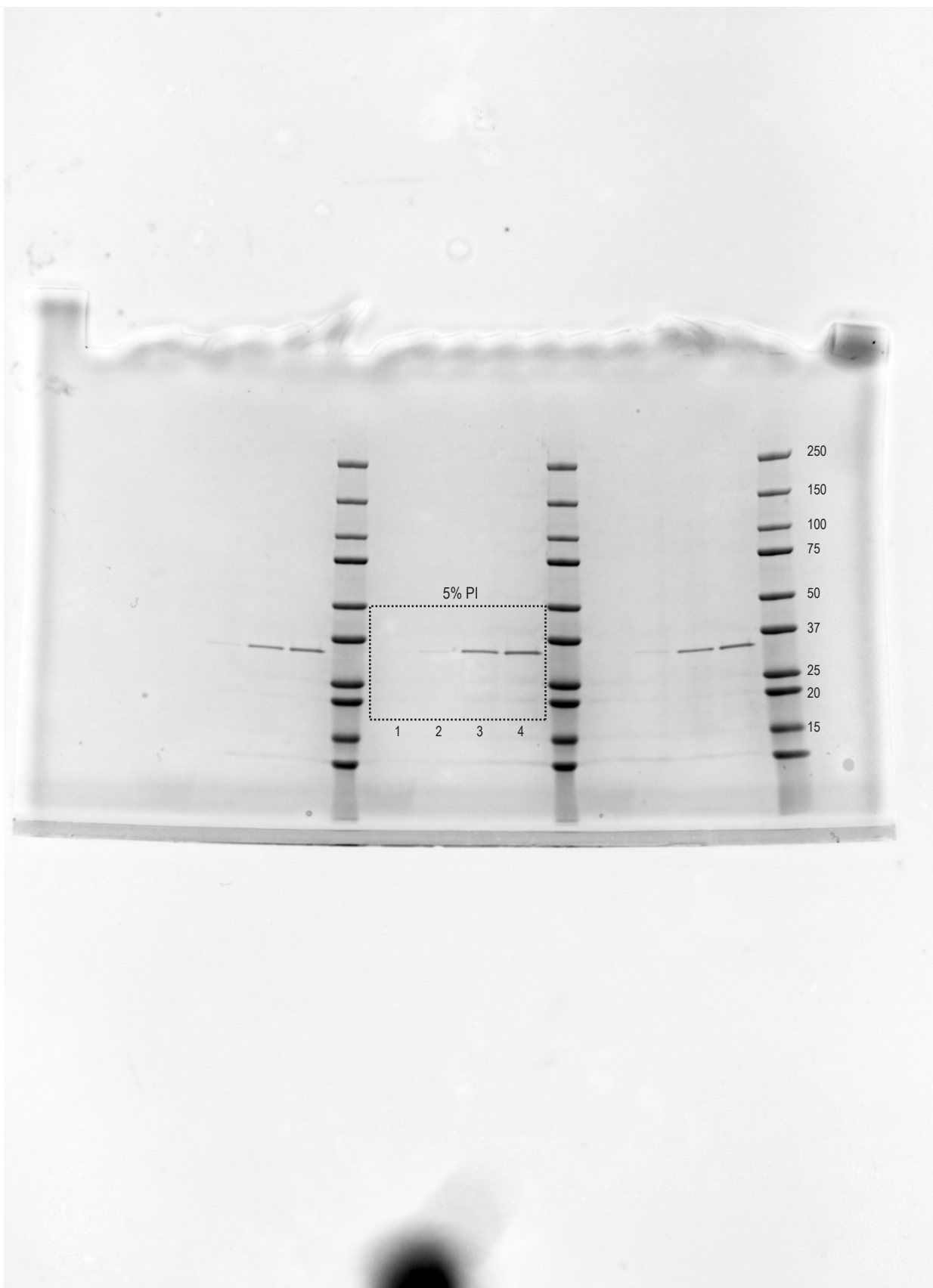

Supplement: SourceData F3 — is the source file for Fig. 3. [file jcb_202409039_sourcedataf3.pdf]

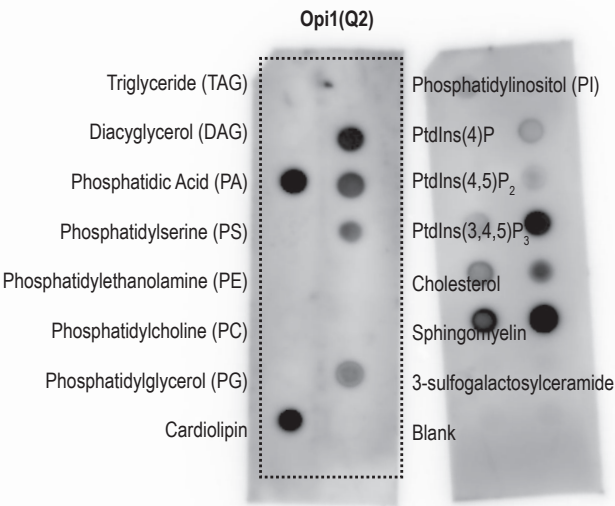

IB: Myc

Pex28-DysF

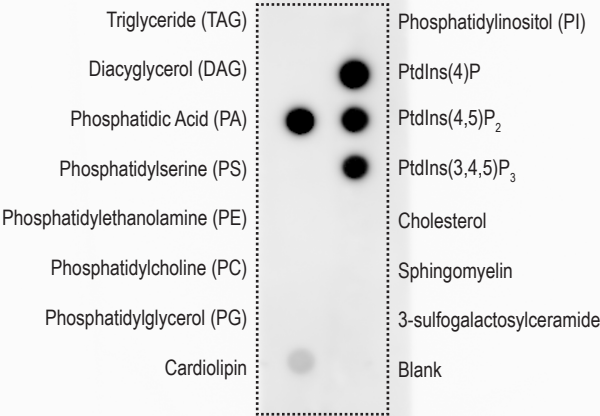

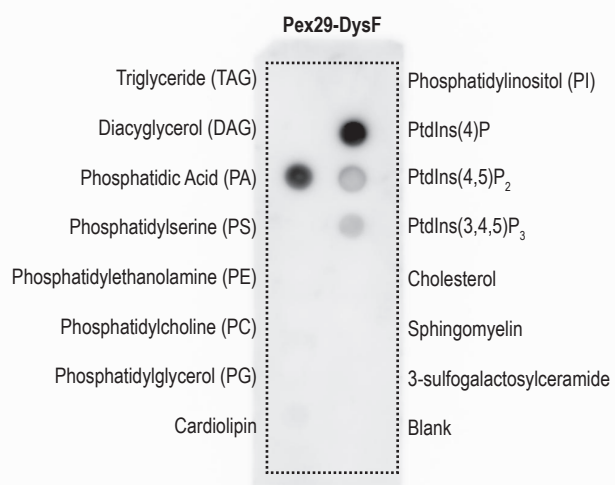

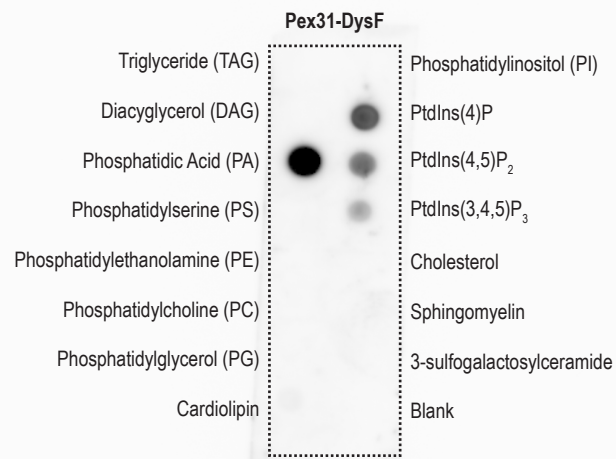

IB: Flag

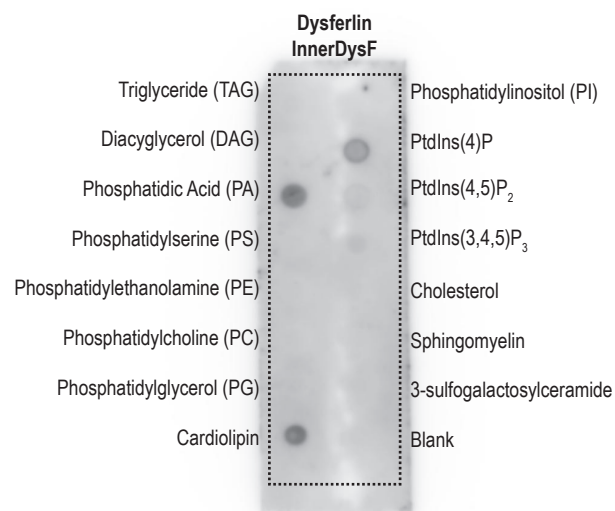

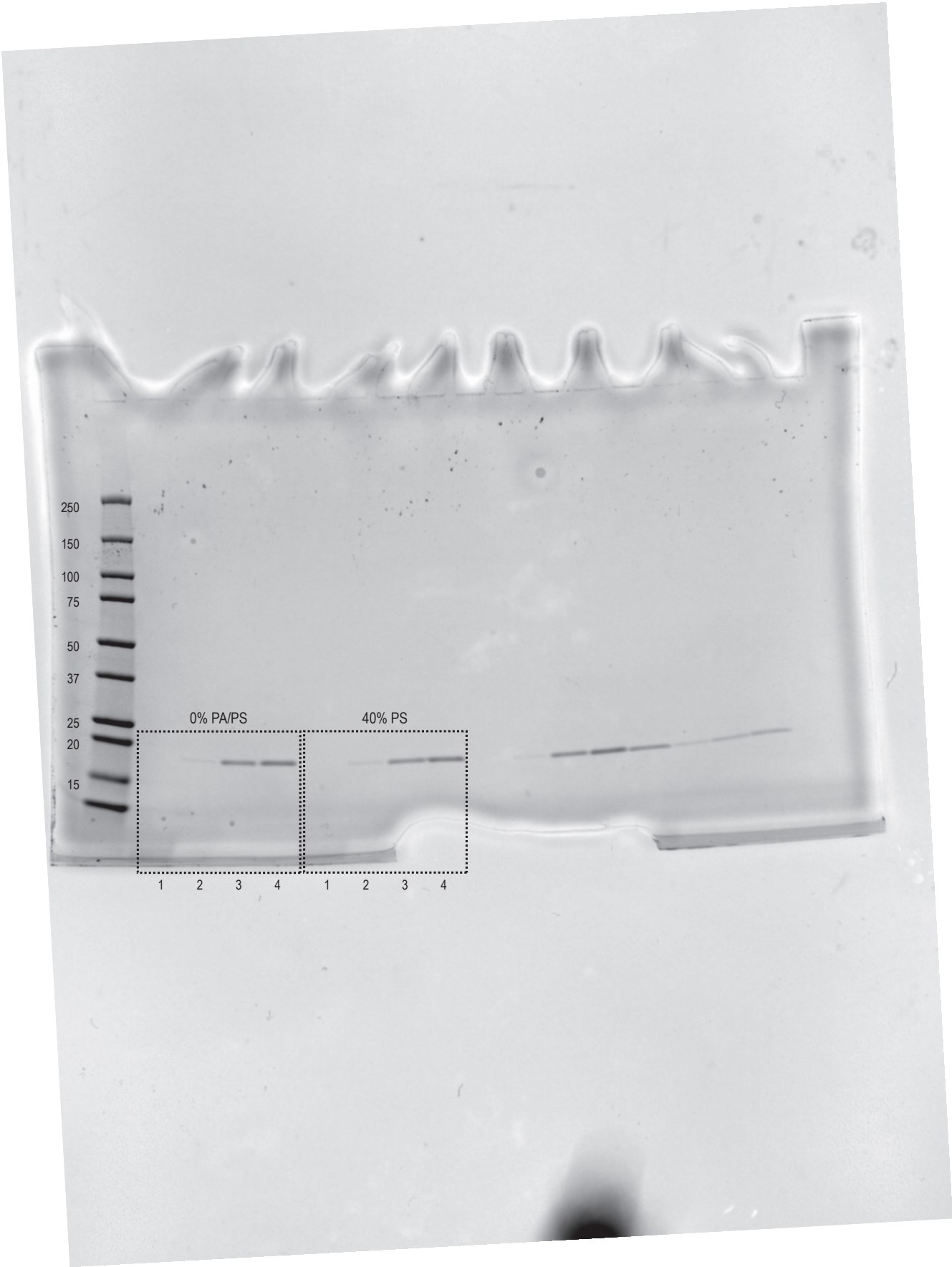

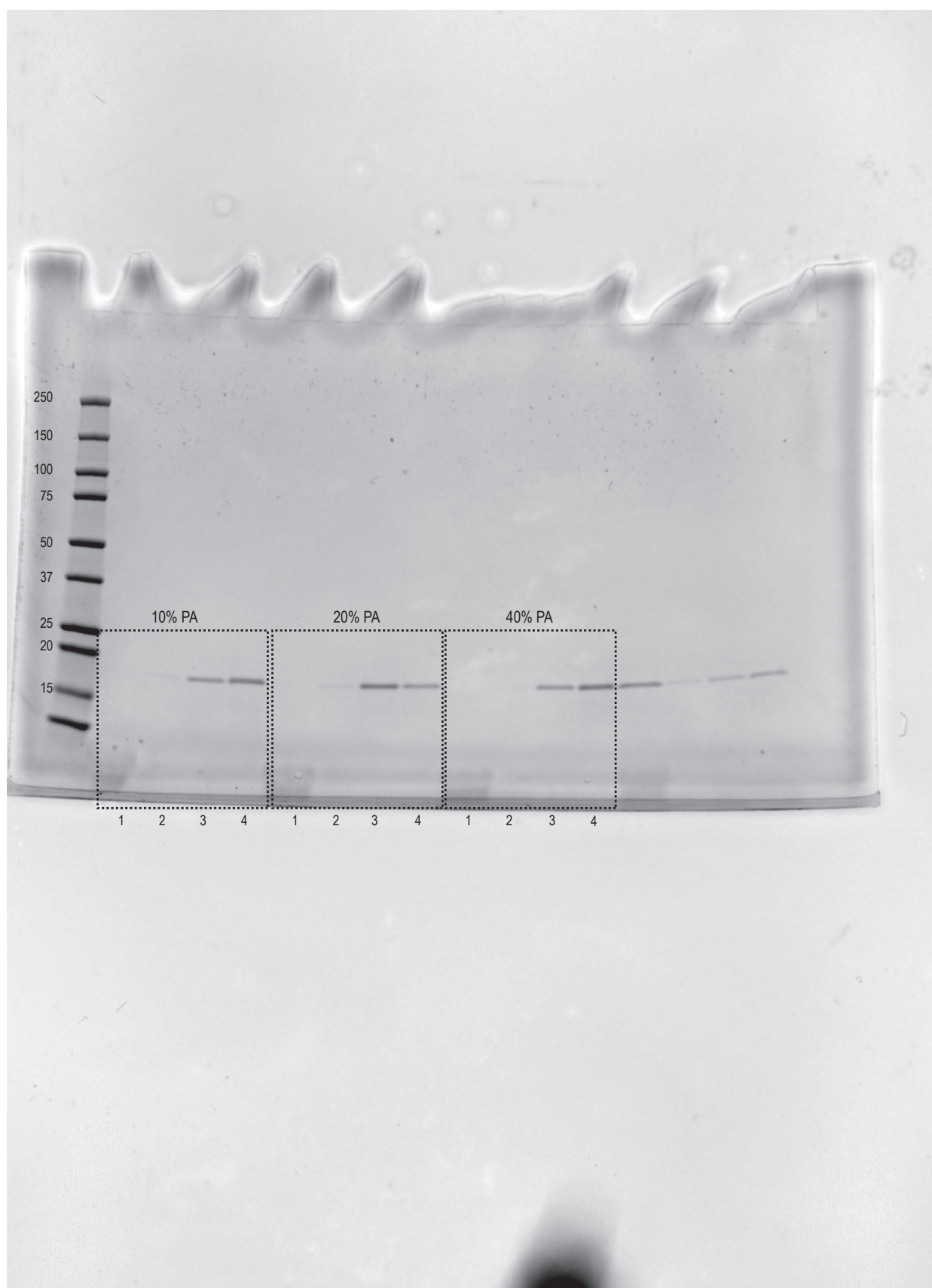

H

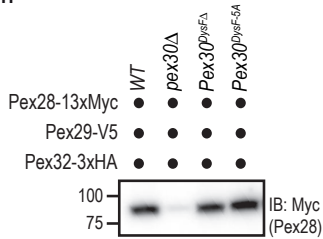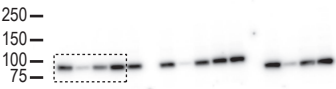

H

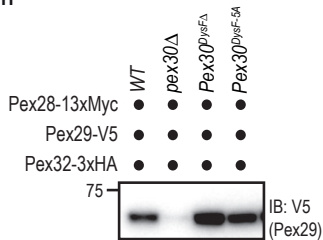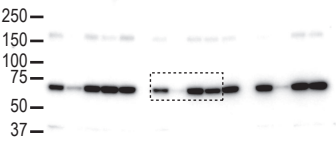

H

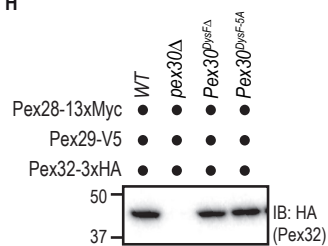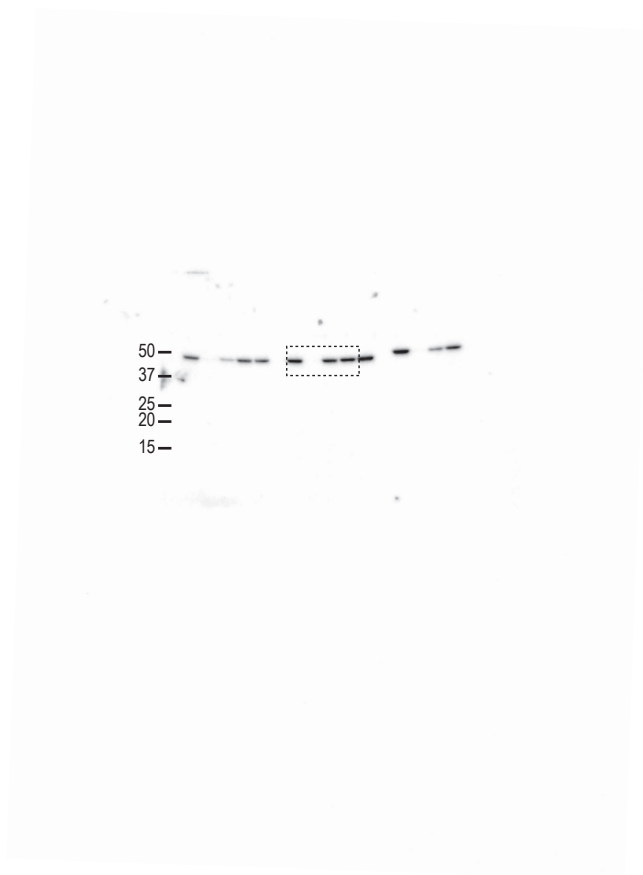

H

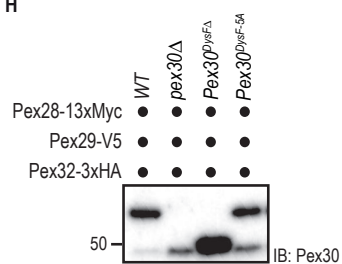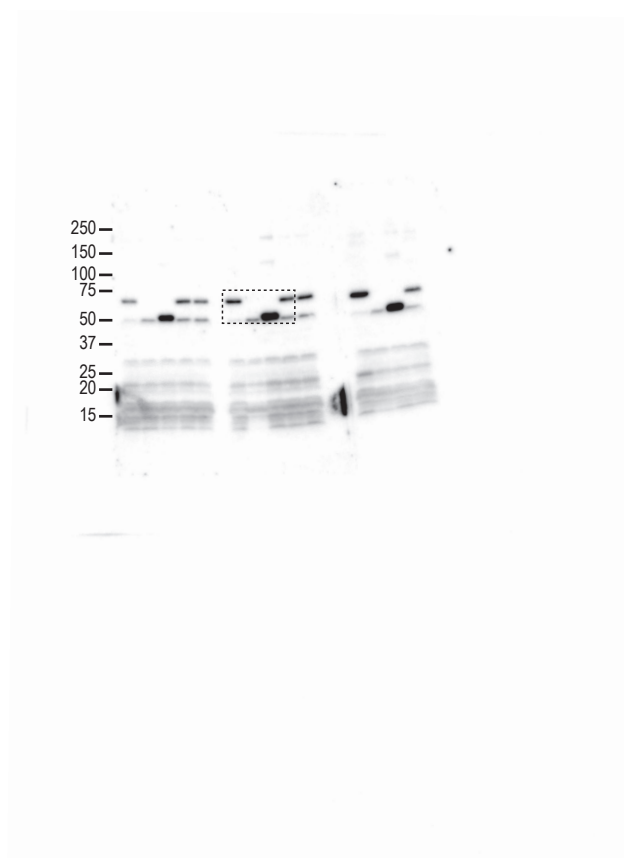

H

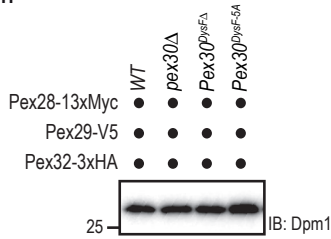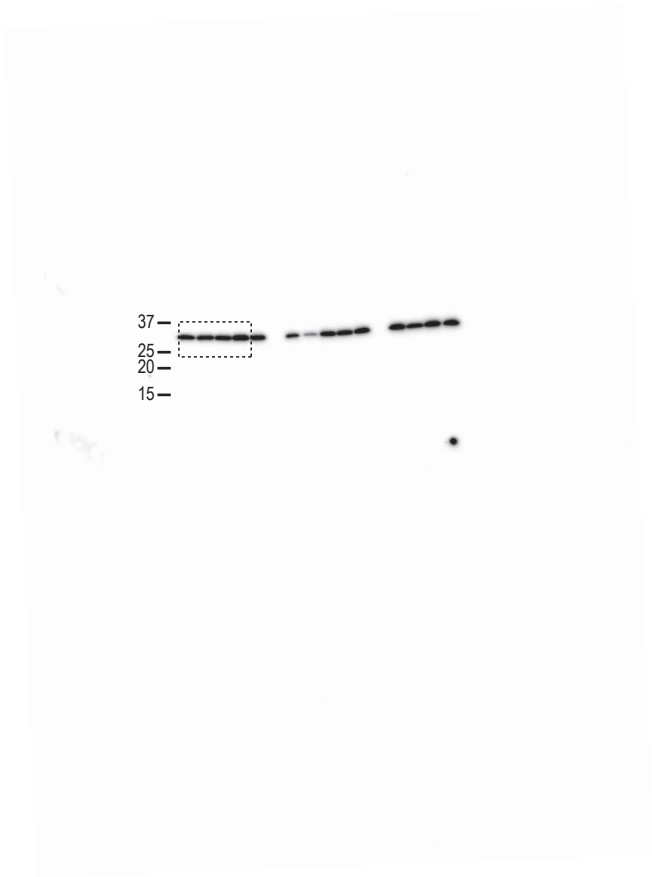

Supplement: SourceData FS3 — is the source file for Fig. S3. [file jcb_202409039_sourcedatafs3.pdf]

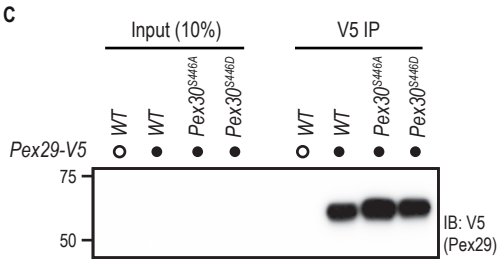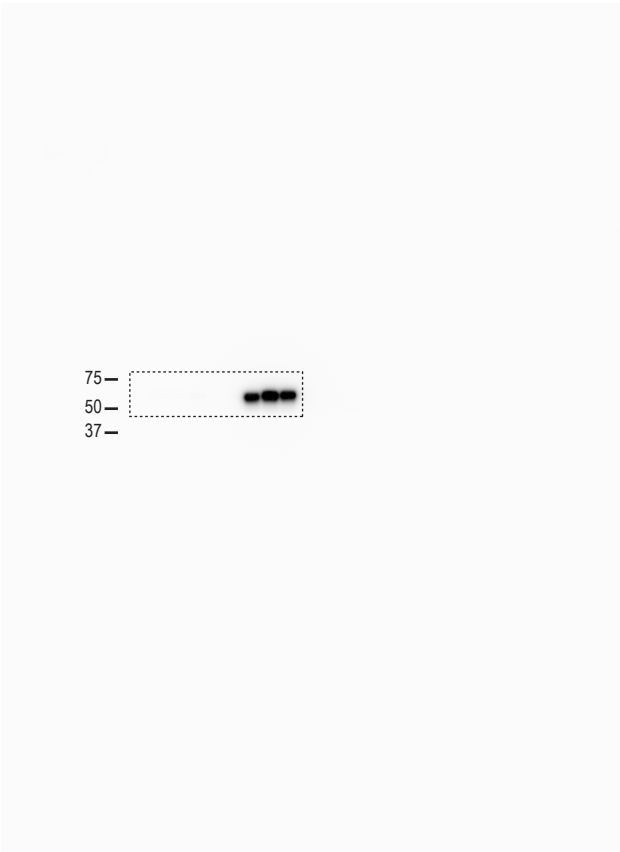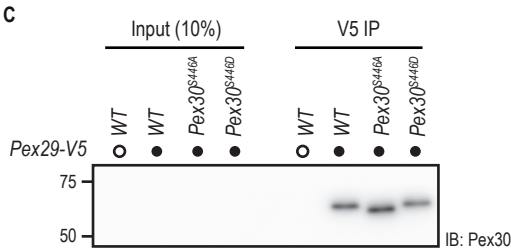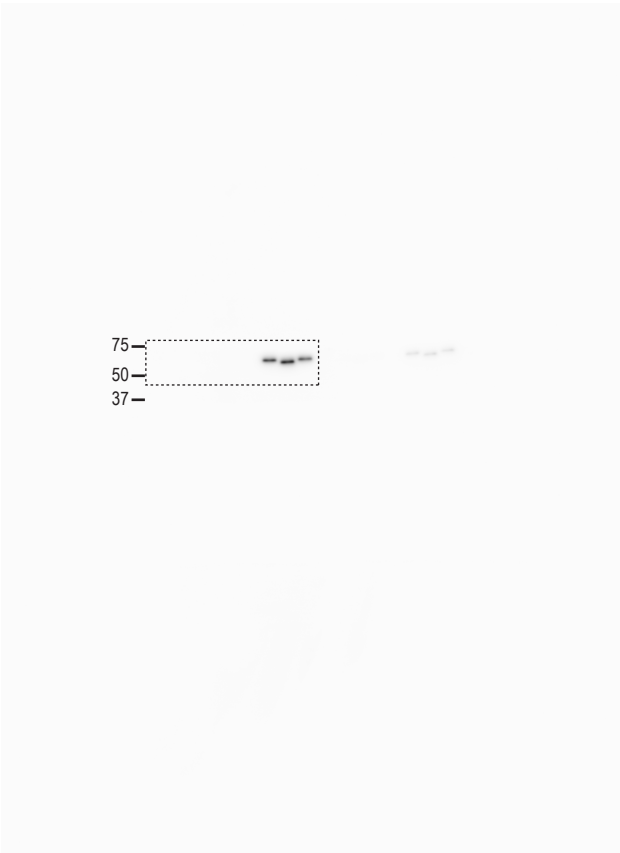

**C**

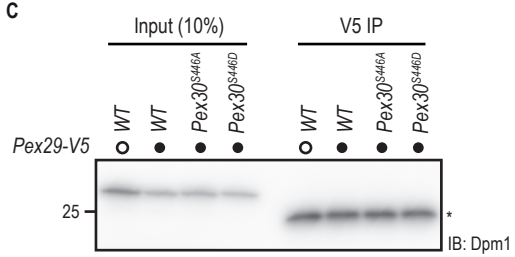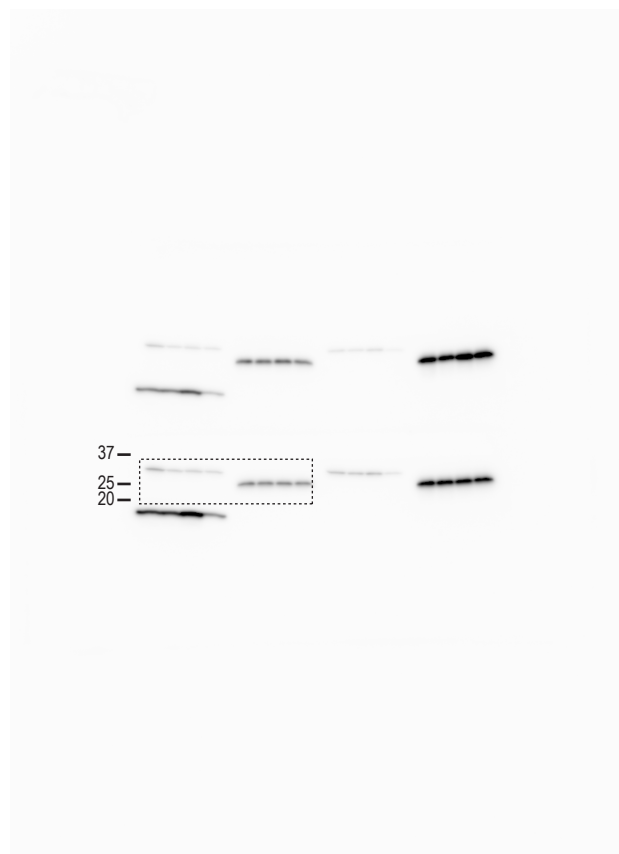

Supplement: SourceData FS5 — is the source file for Fig. S5. [file jcb_202409039_sourcedatafs5.pdf]
