# Supplementary material for: Pex30-dependent membrane contact sites maintain ER lipid homeostasis
Source: J Cell Biol. 2025 May 23;224(7):e202409039. doi: 10.1083/jcb.202409039 (PMC12101078; doi:10.1083/jcb.202409039)
Supplement: SourceData FS2 — is the source file for Fig. S2. [file jcb_202409039_sourcedatafs2.pdf]

SourceDataFS2A

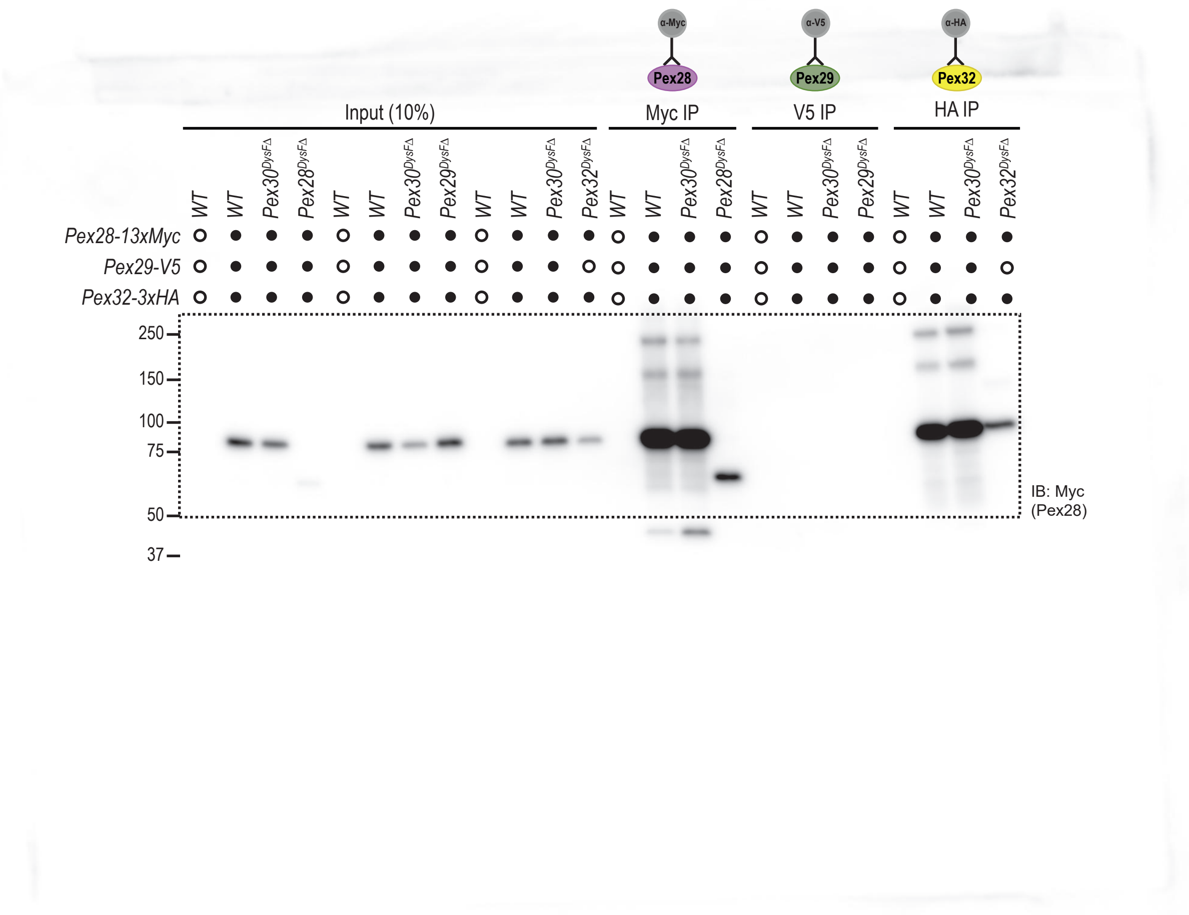

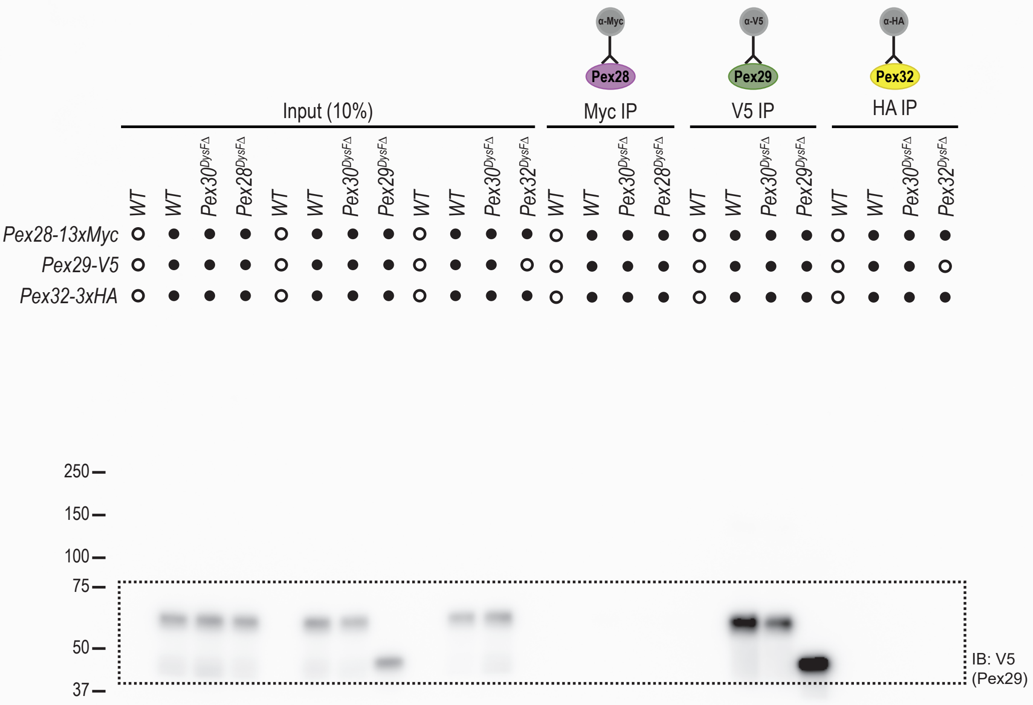

α-Myc
Pex28
Myc IP
α-V5
Pex29
V5 IP
α-HA
Pex32
HA IP
Input (10%)
WT
Pex30DysFΔ
Pex28DysFΔ
Pex29DysFΔ
Pex32DysFΔ
Pex28-13xMyc
Pex29-V5
Pex32-3xHA
250
150
100
75
50
37
IB: V5
(Pex29)

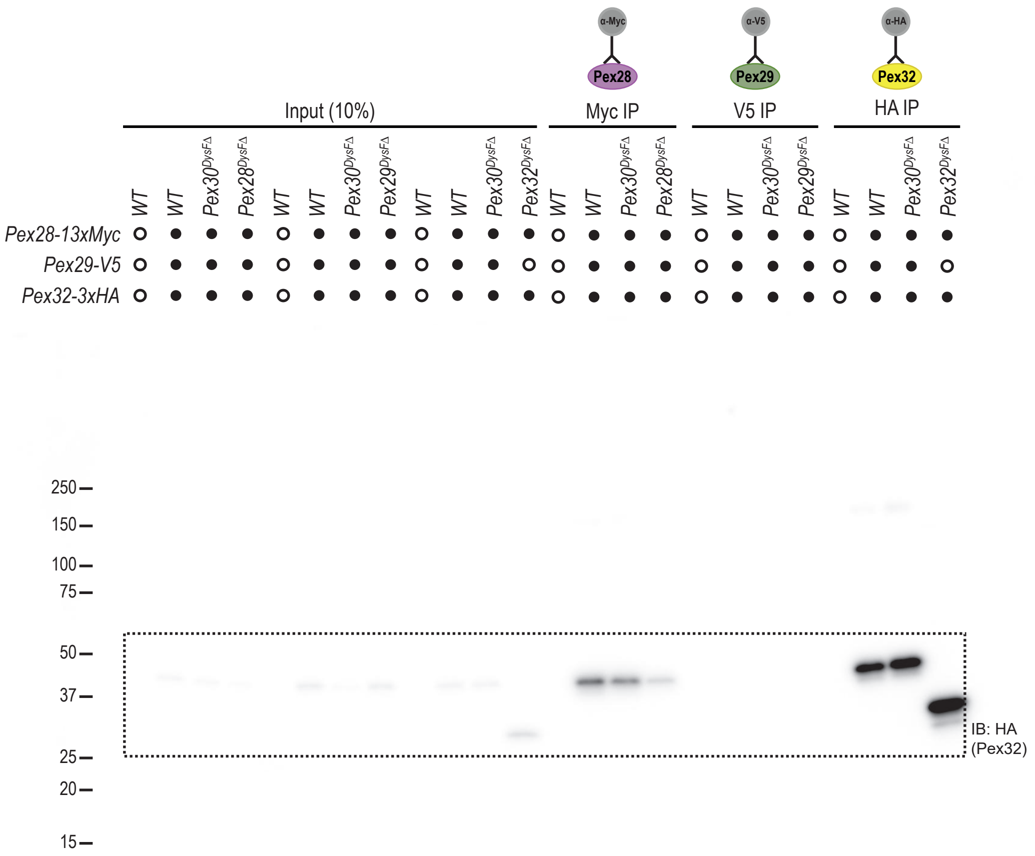
α-Myc
Pex28
α-V5
Pex29
α-HA
Pex32
Input (10%)
Myc IP
V5 IP
HA IP
WT
Pex30DysFΔ
Pex28DysFΔ
Pex29DysFΔ
Pex32DysFΔ
Pex28-13xMyc
Pex29-V5
Pex32-3xHA
250
150
100
75
50
37
25
20
15
IB: HA
(Pex32)

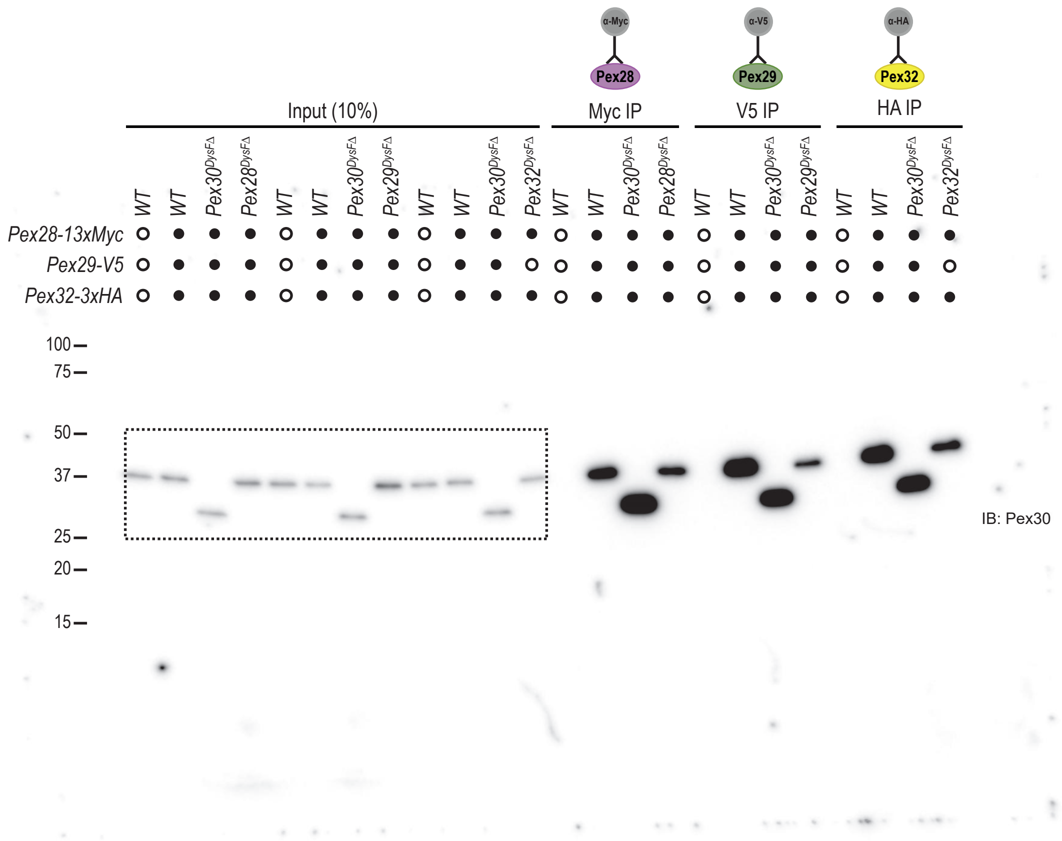

α-Myc
Pex28
α-V5
Pex29
α-HA
Pex32
Input (10%)
Myc IP
V5 IP
HA IP
WT
Pex30DysFΔ
Pex28DysFΔ
Pex29DysFΔ
Pex32DysFΔ
Pex28-13xMyc
Pex29-V5
Pex32-3xHA
100
75
50
37
25
20
15
IB: Pex30

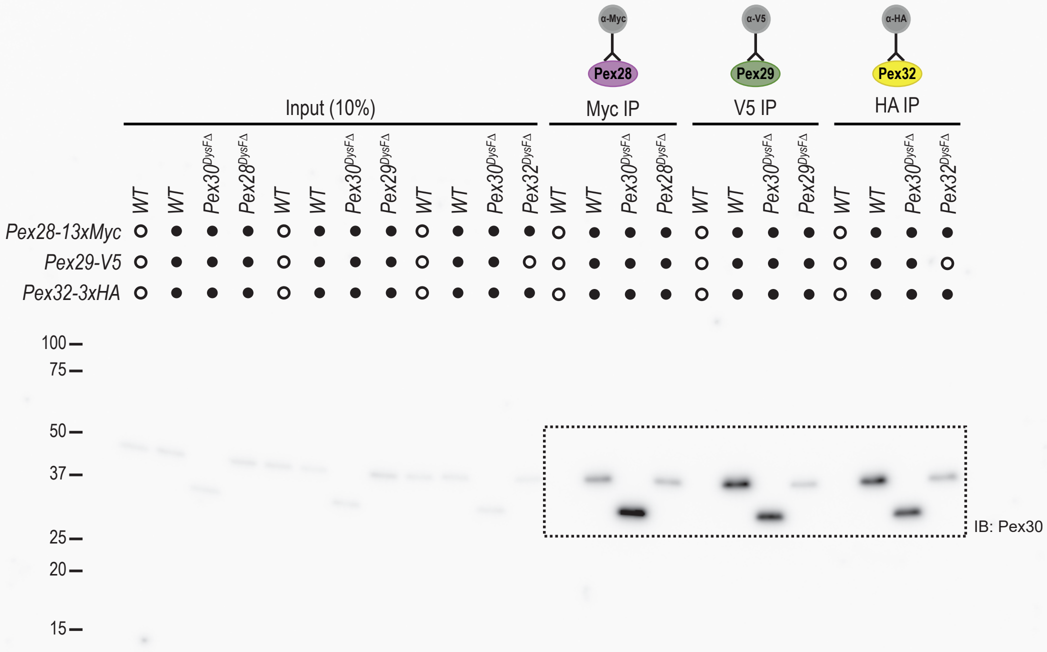

α-Myc
Pex28
α-V5
Pex29
α-HA
Pex32
Input (10%)
Myc IP
V5 IP
HA IP
Pex28-13xMyc
Pex29-V5
Pex32-3xHA
100
75
50
37
25
20
15
IB: Pex30

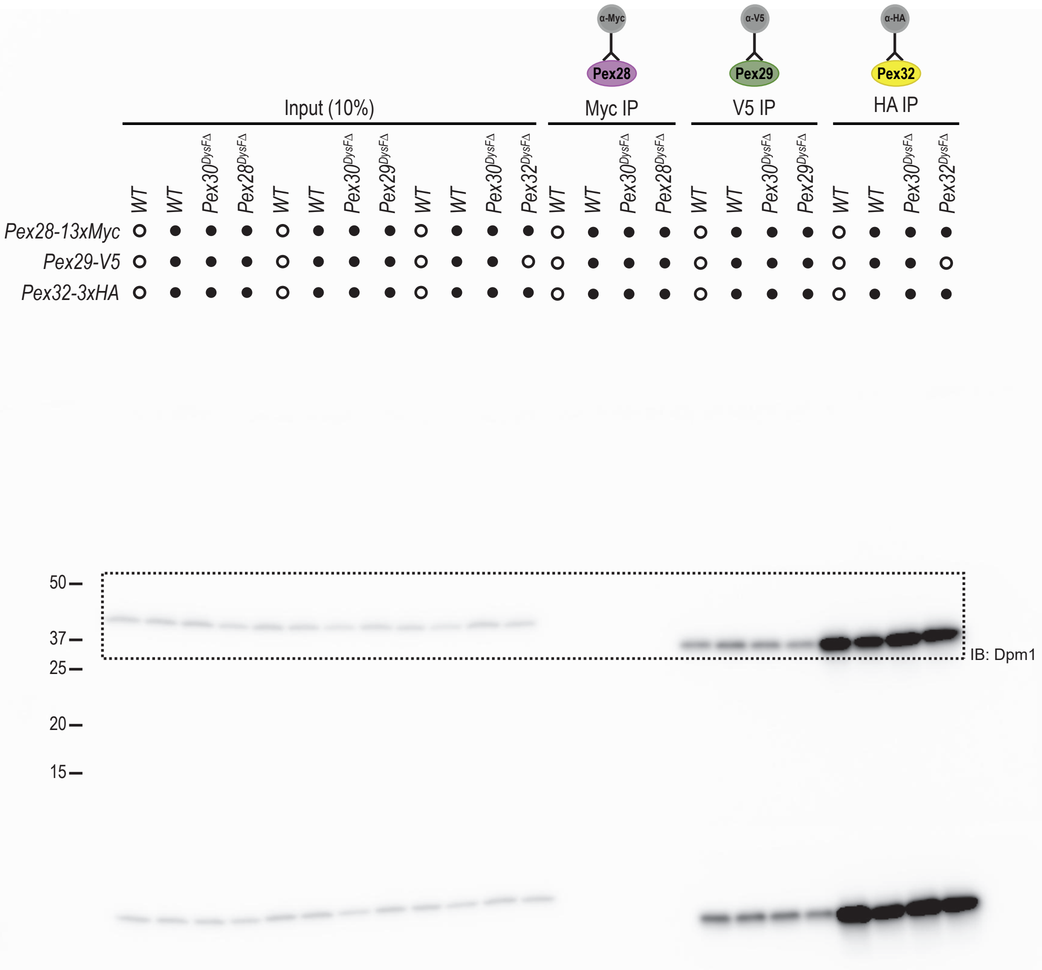
α-Myc
Pex28
α-V5
Pex29
α-HA
Pex32
Input (10%)
Myc IP
V5 IP
HA IP
WT
Pex30DysFΔ
Pex28DysFΔ
Pex29DysFΔ
Pex32DysFΔ
Pex28-13xMyc
Pex29-V5
Pex32-3xHA
50
37
25
20
15
IB: Dpm1
